# Machine learning and magnetic resonance image texture analysis predicts accelerated lung function decline in ex-smokers with and without chronic obstructive pulmonary disease

**DOI:** 10.1117/1.JMI.11.4.046001

**Published:** 2024-07-19

**Authors:** Maksym Sharma, Miranda Kirby, Aaron Fenster, David G. McCormack, Grace Parraga

**Affiliations:** a Robarts Research Institute, London, Ontario, Canada; b Western University, Department of Medical Biophysics, London, Ontario, Canada; c Toronto Metropolitan University, Department of Physics, Toronto, Ontario, Canada; d Western University, Department of Medical Imaging, London, Ontario, Canada; e Western University, Division of Respirology, Department of Medicine, London, Ontario, Canada

**Keywords:** machine learning, hyperpolarized gas magnetic resonance imaging, texture analysis, ex-smokers, chronic obstructive pulmonary disease

## Abstract

**Purpose:**

Our objective was to train machine-learning algorithms on hyperpolarized He3 magnetic resonance imaging (MRI) datasets to generate models of accelerated lung function decline in participants with and without chronic-obstructive-pulmonary-disease. We hypothesized that hyperpolarized gas MRI ventilation, machine-learning, and multivariate modeling could be combined to predict clinically-relevant changes in forced expiratory volume in 1 s (${\mathrm{FEV}}_{1}$) across 3 years.

**Approach:**

Hyperpolarized He3 MRI was acquired using a coronal Cartesian fast gradient recalled echo sequence with a partial echo and segmented using a k-means clustering algorithm. A maximum entropy mask was used to generate a region-of-interest for texture feature extraction using a custom-developed algorithm and the PyRadiomics platform. The principal component and Boruta analyses were used for feature selection. Ensemble-based and single machine-learning classifiers were evaluated using area-under-the-receiver-operator-curve and sensitivity-specificity analysis.

**Results:**

We evaluated 88 ex-smoker participants with 31±7 months follow-up data, 57 of whom (22 females/35 males, 70±9 years) had negligible changes in ${\mathrm{FEV}}_{1}$ and 31 participants (7 females/24 males, 68±9 years) with worsening ${\mathrm{FEV}}_{1}\geq 60\;\mathrm{mL}/\text{year}$. In addition, 3/88 ex-smokers reported a change in smoking status. We generated machine-learning models to predict ${\mathrm{FEV}}_{1}$ decline using demographics, spirometry, and texture features, with the later yielding the highest classification accuracy of 81%. The combined model (trained on all available measurements) achieved the overall best classification accuracy of 82%; however, it was not significantly different from the model trained on MRI texture features alone.

**Conclusion:**

For the first time, we have employed hyperpolarized He3 MRI ventilation texture features and machine-learning to identify ex-smokers with accelerated decline in ${\mathrm{FEV}}_{1}$ with 82% accuracy.

## 1 Introduction

Pulmonary hyperpolarized He3 gas magnetic resonance imaging (MRI) provides a means to quantify ventilation abnormalities using ventilation defect percent (VDP)^1^ that stem from abnormalities in the large and small airways as well as emphysema.^2^ Using forced expiratory volume in 1 s (${\mathrm{FEV}}_{1}$), it is difficult to predict patients with chronic airflow obstruction that will worsen with an accelerated decline in lung function. MRI-VDP measurements were previously shown to progressively worsen in patients with a stable ${\mathrm{FEV}}_{1}$ and predict worse outcomes over short time-periods.^3^^,^^4^ While spirometry measurements of lung function are straightforward and cost-efficient to implement, they do not provide information about the small airways, which are believed to drive COPD pathogenesis.

In contrast, airway structural changes can be evaluated using established quantitative computed tomography (CT) measurements.^5^ Conversely, MRI VDP^1^ provides functional information and has been shown to predict COPD exacerbations^6^ and longitudinal changes in quality of life as well as exercise capacity.^3^ Recent studies have shown that CT radiomics features are associated with lung function in COPD,^7^ emphysema severity,^8^ and provide complementary information to established quantitative CT measurements.^9^^,^^10^ Despite these advantages, current predictive models of COPD progression are usually based on clinical measurements but none incorporate information derived from pulmonary CT or magnetic resonance imaging (MRI).^11^

Texture analysis provides a unique opportunity to reveal and quantify hyperpolarized He3 MRI ventilation patchiness. Several recent investigations in COPD^8^^,^^10^^,^^12^ have clearly demonstrated the advantages of using radiomics approaches on CT images. Since binary VDP measurements do not exploit the full spectrum of information and spatial content that is inherent to hyperpolarized gas MRI, our main objective was to develop a texture-based machine learning model to identify ventilation features that can predict patients with an accelerated annual ${\mathrm{FEV}}_{1}$ decline. Our secondary objective was to generate novel measurements of MRI ventilation heterogeneity and test their performance at predicting accelerated ${\mathrm{FEV}}_{1}$ decline.

In COPD, rapid decliners have been previously defined as patients with a decline in ${\mathrm{FEV}}_{1}\geq 40$^13^^,^^14^ or ≥60  mL/year.^10^^,^^15^^–^^18^ In general, the annual ${\mathrm{FEV}}_{1}$ decline is larger in patients with mild COPD stages and less pronounced airflow limitation.^16^^,^^18^ Therefore, we tested multiple single and ensemble classifiers to determine the best model for predicting COPD patients who would experience an ${\mathrm{FEV}}_{1}$ decline ≥60  mL/year, over a 3-year period. Such predictive models may serve as tools for an early detection of rapidly progressing patients and facilitate early treatment options for this subgroup of patients that are at a higher risk of progressing to a greater disease severity.

## 2 Materials and Methods

### 2.1 Study Design and Participants

**Fig. 1:**
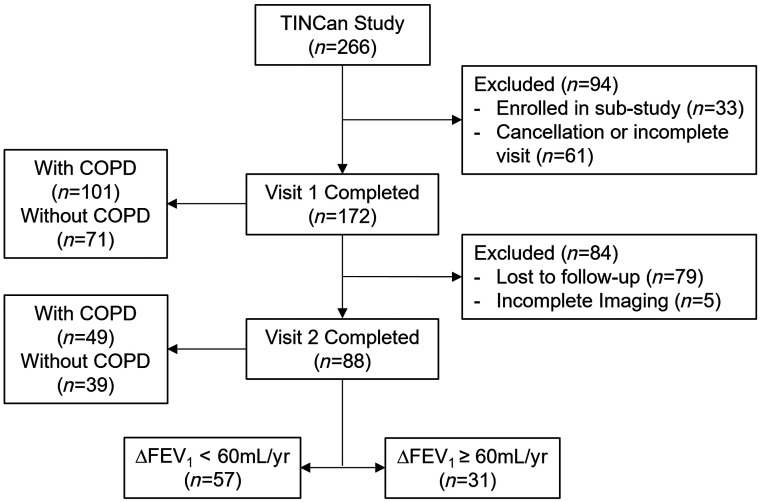
CONSORT flow diagram. Of the 266 participants enrolled in the TINCan study, 33 were enrolled in a sub-study and 61 either cancelled or did not complete all required tests during visit 1. Of the 172 participants that completed visit 1, 79 participants did not complete a 3-year follow-up visit 2 and 5 had artifacts present in their images, which were excluded from further analysis. Eighty-eight ex-smoker participants with visit 1 and visit 2 data were analyzed in this study, of which 31 had $\Delta {\mathrm{FEV}}_{1}\geq 60\;\mathrm{mL}/\text{year}$ and 57 had $\Delta {\mathrm{FEV}}_{1}<60\;\mathrm{mL}/\text{year}$.

All participants provided informed written consent to a study protocol approved by a local research ethics board and in compliance with the Health Canada approved and registered protocol^19^ (clinicaltrials.gov NCT02279329, Institutional Review Board IRB00000940). Inclusion criteria were a history of cigarette smoking >10 pack years, age between 50 and 85 years at baseline. Ex-smoker participants were included who had ceased smoking ≥1 year prior to the study visit, with no cut-off in terms of smoking cessation. COPD subjects were classified according to the Global Initiative for Chronic Obstructive Lung Disease (GOLD) grades.^20^ Participants also completed a longitudinal follow-up visit at 24±6 months after the baseline visit.^19^ Participants that reported a change in smoking status from ex-smokers to current smokers at follow-up visit were included in the analysis. The CONSORT diagram for the Thoracic Imaging Network of Canada (TINCan) study cohort participants is depicted in Fig. 1.

### 2.2 Pulmonary Function Tests and Image Acquisition

Spirometry, plethysmography, and the diffusing capacity of the lungs for carbon monoxide (${\mathrm{DL}}_{\mathrm{CO}}$) were measured according to the American Thoracic Society/European Respiratory Society guidelines^21^ using a whole-body plethysmography system (MedGraphics Corporation, St Paul, Minnesota, United States) and attached gas analyzer.^22^ COPD was defined as post-bronchodilator spirometry according to the GOLD criteria.^20^ The 6MWD^23^ test and St. George’s Respiratory Questionnaire (SGRQ)^24^ were administered under supervision.

Anatomic proton (H1) and hyperpolarized He3 MR images were acquired using a whole-body 3.0 Tesla Discovery MR750 system (GE Healthcare, Milwaukee, Wisconsin, United States), a whole-body radiofrequency coil and a fast gradient recalled echo (FGRE) sequence with a partial echo implementation, with acquisition parameters as previously described.^25^ Hyperpolarized He3 MRI was acquired using a linear bird-cage transmit/receive chest coil (RAPID Biomedical GmbH, Wuerzburg, Germany). A turn-key system (HeliSpin™, Polarean Inc, Durham, North Carolina, United States) was used to polarize He3 gas to 30% to 40% and doses (5  mL/kg body weight) diluted with $N_{2}$ were administered in 1.0 L Tedlar^®^ bags. Hyperpolarized He3 MRI diffusion-weighted imaging was performed using a 2D multi-slice fast gradient-echo method, as previously described,^25^ during breath-hold for acquisition of two interleaved images with and without additional diffusion sensitization with $b=1.6\;\sec/{\mathrm{cm}}^{2}$ (maximum gradient amplitude [G] = 1.94 G/cm, rise and fall-time = 0.5 ms, gradient duration = 0.46 ms, and diffusion time = 1.46 ms).^19^ Pulmonary function data and imaging were acquired during both baseline and follow-up visits.

### 2.3 Image Analysis and Proposed Algorithm

Baseline and follow-up visit H1 and He3 MR images were processed by a single observer where the thoracic cavity was segmented from the H1 images using a seeded region-growing algorithm, and the He3 ventilation region was segmented using k-means clustering.^1^ The generated maximum entropy mask was then applied to identify the ventilated region-of-interest (ROI) for feature extraction. Diffusion-weighted images were automatically processed to generate apparent diffusion coefficient (ADC) values and images, as previously described.^22^

MRI VDP was generated using a semi-automated segmentation approach, as previously described.^1^ Ventilation defect cluster percent (VDCP), which is the sum of ventilation-defect cluster volume normalized to the total lung volume, and defect cluster sizes were generated by an automated in-house custom-developed algorithm, as described in the prior proceedings paper.^26^ Briefly, the proposed cluster algorithm iteratively segments unventilated MRI volumes until the maximum sphere fit (or multiple spheres of the same size) within the unventilated volume is identified. This sphere volume(s) is then removed from the non-ventilated region and the process is iteratively repeated until the non-ventilated region is filled by spheres. This is similar in approach to sphere packing previously investigated for radio-surgical treatment planning.^27^

The approach was implemented using a naïve greedy algorithm where $S=[b_{1},b_{2},\ldots ,b_{n}]$ is a set with n elements, where each element $b_{n}=B_{n}(r,l)$ is an open sphere of radius r at locations $\vec{l}$. Determining the required minimum number of spheres of unequal sizes resulted in the following minimization problem

$$\underset{S}{\min}\;\{{\left\|S\right\|}_{0}\}:\;S\in R^{n},$$ (1)

where the cardinality of the set S and ∀  (b∈S)  ∃  (r,l) is minimized. To ensure that the spheres completely fill the unventilated region-of-interest R and are within the thoracic cavity, several constraints were implemented. First, the intersection between the region that is being filled with spheres (R) and the spheres (b) was set to b. Furthermore, to prevent spheres from overlapping, the overlap between two spheres (b and $b^{\prime }$) was fixed to result in a null set

$$b\cap R=b\;\&\;b\cap b^{\prime }=Ø.$$ (2)

A volume constraint was also imposed such that the total volume of spheres was equal to the volume of the specified unventilated region R

$$\underset{b\in S}{\sum }V(b)=V(R).$$ (3)

The specified regions were filled with spheres, where the minimum sphere diameter was equal to one voxel ($5\times 5\times 5\;{\mathrm{mm}}^{3}$) such that the total volume of spheres with diameter equal to one voxel was equal to be the total residual volume not clustered into large (>one voxel) sphere sizes. To further simplify the problem, there were no location constraints on the sphere spatial position.

**Fig. 2:**
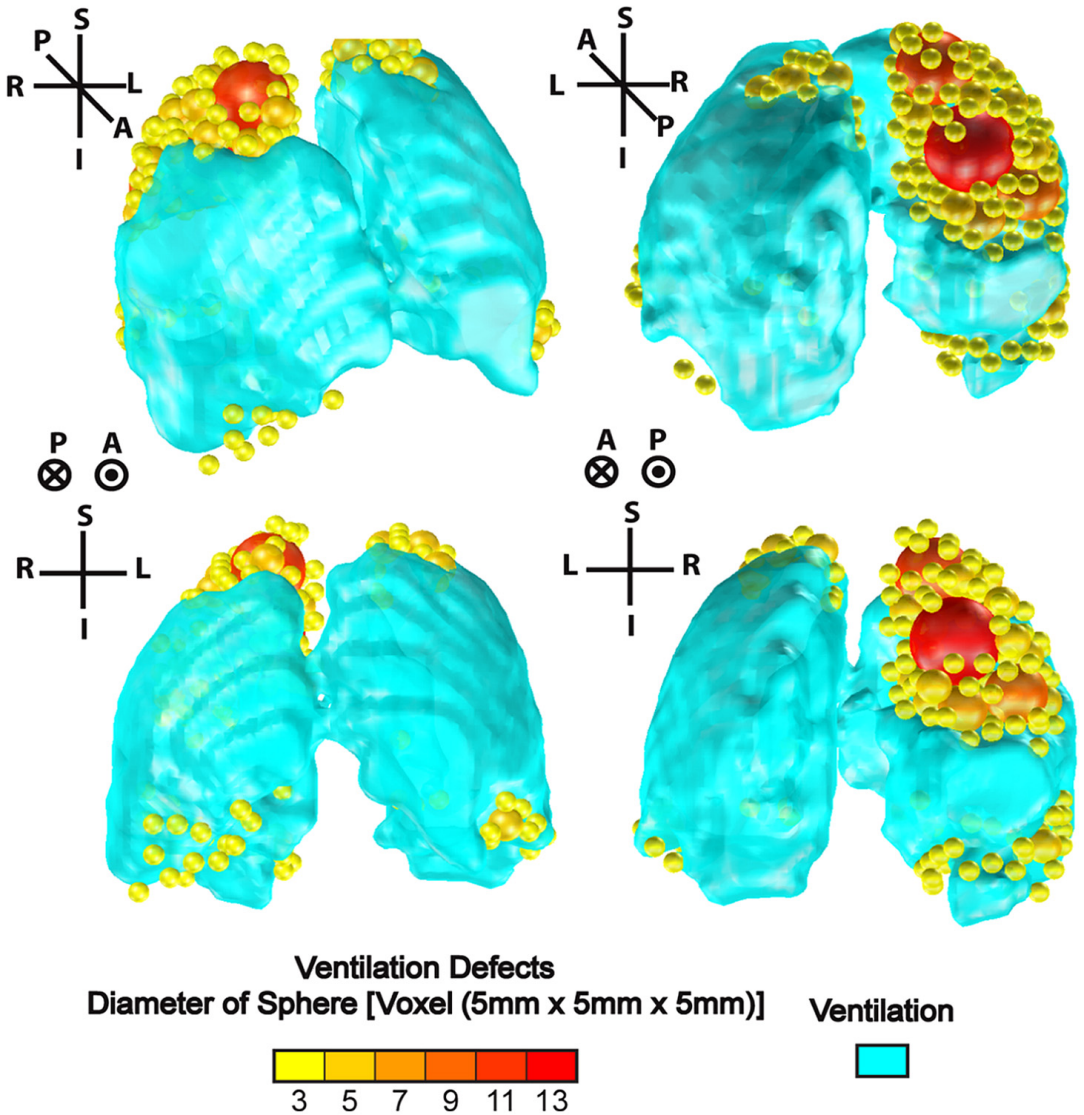
Ventilation defect cluster volume output from custom-developed algorithm. A representative 66-year old male ex-smoker participant with COPD: ${\mathrm{FEV}}_{1}=1.93\;L$, VDP = 25%, $\Delta {\mathrm{FEV}}_{1}=-0.22\;L$, and ΔVDP=1% between visits. Three-dimensional isotropic ventilation volume shown in cyan and ventilation defects represented by different sphere sizes ranging from small (yellow = sphere diameters of 3 to 5 voxels) to large (red = sphere diameters of 9 to 13 voxels).

We used MATLAB R2021a (MathWorks) to solve the minimization problem and generated VDCP to calculate cluster-defect-diameter voxel size one (CDD1) or the number of small ventilation defects, which is the cumulative number of defect clusters of one voxel, as shown in Fig. 2. Low ventilation cluster slopes were also calculated based on the log–log relationship between the cumulative number of spheres and cluster size.^28^^,^^29^

Further texture feature extraction was conducted using an open-source PyRadiomics software, detailed in the next section. Unlike the proposed algorithm that analyzes the unventilated region, texture features were extracted from the inhaled hyperpolarized gas distribution within the thoracic cavity.

### 2.4 Feature Extraction and Selection Pipeline

**Fig. 3:**
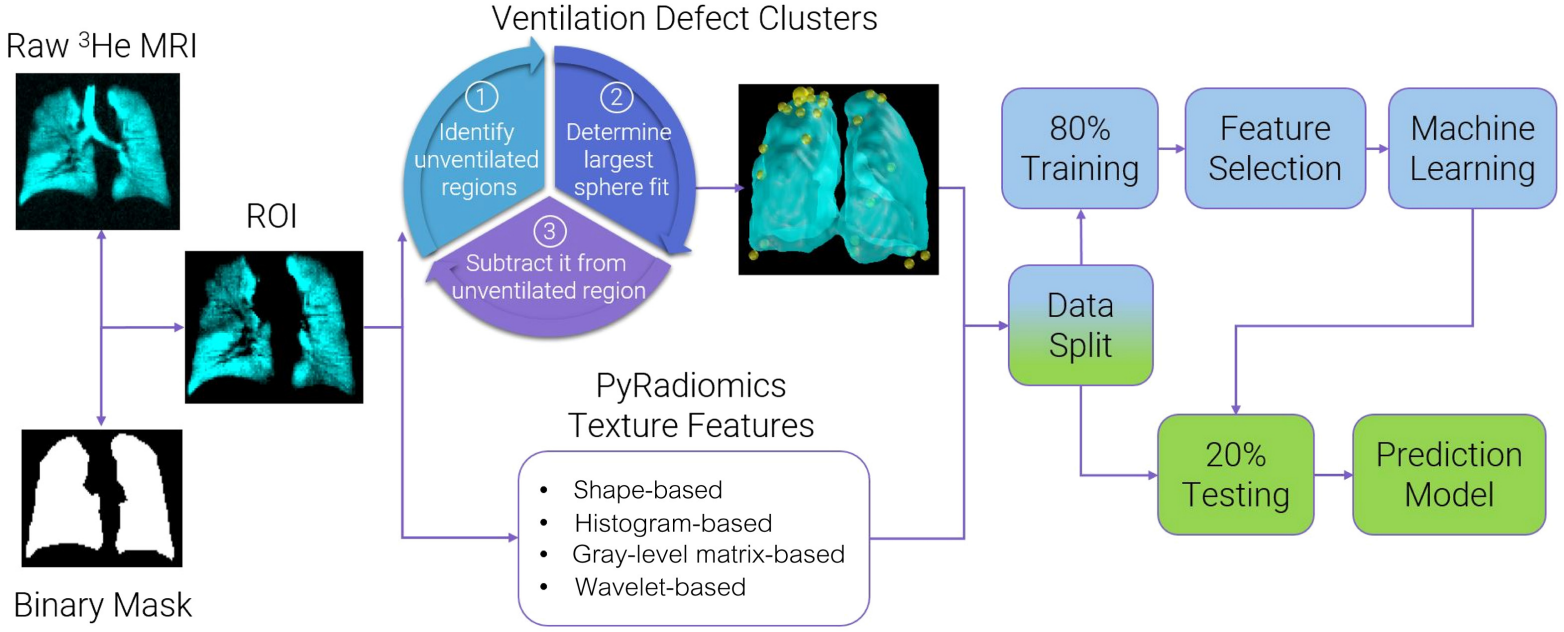
Image processing and model generation pipeline. Hyperpolarized noble gas MR images were analyzed using custom-developed algorithms to calculate ventilation defect clusters and texture features. Raw MR images and corresponding binary lung masks were used to generate the regions-of-interest (ROI), which were analyzed via the PyRadiomics platform to calculate shape and first- and higher-order texture features. Feature selection was performed using the training set, combined with PCA and Boruta analysis. Classification learner application was used for model generation, which was trained using a fivefold cross-validation scheme, with 20% of data reserved for the testing set.

The complete pipeline for processing the baseline and follow-up visit images is summarized in Fig. 3. First, we generate a maximum entropy mask by segmenting the He3 ventilation image as previously described.^1^ We then use a custom-developed algorithm to calculate the ventilation defect clusters, described above, and the PyRadiomics platform^30^ for extracting texture features. Texture features were calculated from gray-level histograms and matrices generated from the ROI of the original image.

We generated first-order texture features from the gray-level histograms and also evaluated the texture features calculated from run-length, gap-length, co-occurrence, size-zone, dependence, and neighborhood gray tone matrices using the PyRadiomics open-sourced platform (version 2.2.0) in Python environment (version 3.7.5).^30^ Image processing filters were also applied for the extraction of wavelet band-pass filtering texture features. This further quadrupled the number of extracted features due to permutations of high-pass and low-pass filters for wavelet decomposition, resulting in 376 additional MRI texture features. Low-pass filtering in both directions (LL) assesses the lowest frequencies, low-pass filtering followed by high-pass filtering (LH) assesses horizontal edges, high-pass filtering followed by low-pass filtering (HL) assesses vertical edges and high-pass filtering in both directions (HH) assesses diagonal details.^31^

The resulting data and 496 extracted features were randomly assigned into training and testing sets with 80%/20% data split. Participants were randomly divided into five groups and models were trained using the training set and a fivefold cross-validation scheme. Therefore, training was performed during five iterations, whereby for each iteration, the model was trained on four groups and validated on one group. Classification learner application (MATLAB R2021a) was used for generating all the models using the training dataset, with 20% of unseen data reserved as the final testing set to determine model accuracy. Feature selection was performed on the training set using principal component analysis (PCA) and Boruta analysis to rank and determine non-redundant texture features significantly contributing to the predictive power of the machine learning models.

PCA begins by standardizing the data and calculating the covariance matrix, which describes the relationships between features. This matrix is decomposed into eigenvectors and eigenvalues, representing the directions of maximum variance and their magnitudes, respectively. The top eigenvectors, known as principal components, are selected based on their corresponding eigenvalues to capture the most variance in the data. These principal components are used to transform the original data into a lower-dimensional space while preserving as much variance as possible. Boruta analysis is a feature selection technique designed to identify pertinent variables amidst high-dimensional datasets and used a two-step correction for multiple testing. Leveraging a random forest algorithm in conjunction with shadow feature creation, Boruta evaluates the importance of predictor variables iteratively, distinguishing between genuine predictors and random noise. This methodological approach enhances model interpretability and reduces the risk of overfitting. After creating shadow features using the original features, they are concatenated with the original dataset and used for training the random forest classifier. Feature importance is computed for the highest-rated shadow features and all original features that are more important than the corresponding shadow features are kept. This is repeated for multiple iterations, keeping track of the most important features using z-score statistics to determine the final subset of features [number of trees in the forest = 200, maximum iterations = 300, maximum tree depth = 10 (branches), percentage of shadow feature threshold = 95%, and alpha-level = 0.05]. We utilized all wavelet band-pass filtering features that were available in the PyRadiomics platform (version 2.2.0), with the detailed mathematical descriptions of all the selected and extracted texture features provided in Table 3 and Table S1 in the Supplementary Material.^30^

### 2.5 Machine learning and Statistical Analysis

Once all the features and parameters were selected, machine learning models were generated based on (1) demographic measurements alone, (2) spirometry measurements alone, (3) imaging and texture measurements alone, and (4) combination of all available measurements. Fivefold cross-validation training was performed using several machine learning algorithms, including single classifiers and ensemble classifiers, to determine the best model for identifying accelerated disease progression. The data were standardized and hyper-parameter optimization was performed through MATLAB R2021a (Classification Learner App) for each model individually. We compared the performance of multiple machine learning algorithms, including variations of: logistic regression, Naïve Bayes,^32^ support vector machines (SVMs),^33^ decision trees,^34^ K-nearest neighbors (KNNs),^35^ and four ensemble-classifiers: bagged and boosted trees,^36^ subspace discriminant,^37^ subspace K-nearest-neighbors,^37^ and random under-sampling boosted (RUSBoosted) trees. Model performance was evaluated using the mean cross-validation area under the receiver-operator curve (AUC) from training, as well as sensitivity, specificity, and F1-score using the test set and models’ confusion matrix. The DeLong’s test was used to compare the performance of all machine learning models.^38^

Statistical analysis was performed using SPSS Statistics v28.0 (IBM Statistics, Armonk, New York, United States). Shapiro–Wilk tests were used to determine the normality of the data and non-parametric tests were performed for data that were not normal. Differences between subject groups were determined using analysis of variance with post-hoc analysis using the Benjamini–Hochberg correction. The relationship between measurements was determined using Pearson and Spearman coefficients for parametric and non-parametric data, respectively. Holm–Bonferroni correction was applied for multiple comparison tests for the selected texture features. Results were considered significant when the probability of two-tailed type I error was less than 5% (p<0.05).

## 3 Results

A CONSORT diagram provided in Fig. 1 shows that 266 ex-smokers were enrolled and 94 were excluded from analysis due to enrollment in another sub-study (n=33) and due to cancellation or not completing all required tests per protocol (n=61). Of the 172 participants that completed visit 1, 79 participants did not complete a 3-year follow-up visit and 5 had poor image quality and were excluded from further analysis.

### 3.1 Participant Demographics

We evaluated 88 ex-smoker participants, of which 49 had spirometry evidence of COPD and 39 with no spirometry evidence of COPD. As shown in Table 1, 57 participants (22 females/35 males, 70±9 years) demonstrated a smaller ${\mathrm{FEV}}_{1}$ decline (<60  mL/year) and 31 participants (7 females/24 males, 68±9 years) demonstrated a rapid decline in ${\mathrm{FEV}}_{1}$ (≥60  mL/year), between baseline and follow-up visit 31±7 months later. At baseline, there were significant differences only in forced vital capacity (FVC) between subgroups [FVC (L) p=0.003 and FVC (${\%}_{\mathrm{pred}}$) p=0.03, respectively]. As summarized in Table 2, these subgroups did not have any statistically significant differences at follow-up.

**Table 1:** Baseline participant demographics and pulmonary function measurements.

Parameter mean (±SD)	All participants (n=88)	$\Delta {\mathrm{FEV}}_{1}<60\;\mathrm{mL}/\mathrm{yr}$ (n=57)	$\Delta {\mathrm{FEV}}_{1}\geq 60\;\mathrm{mL}/\mathrm{yr}$ (n=31)	p-value
Age	69 (9)	70 (9)	68 (9)	0.2
Female n (%)	29 (33)	22 (39)	7 (23)	0.1
Height (m)	169 (8)	168 (8)	171 (7)	0.2
BMI ($\mathrm{kg}/m^{2}$)	28 (4)	28 (4)	29 (5)	0.4
${\mathrm{SpO}}_{2}$ (%)	96 (3)	95 (4)	96 (2)	0.8
Pack years	36 (26)	37 (26)	35 (21)	0.8
Years since quit	15 (13)	14 (14)	16 (13)	0.7
Pulmonary function and QoL				
${\mathrm{FEV}}_{1}$ (L)	2.3 (0.8)	2.2 (0.8)	2.5 (0.8)	0.1
${\mathrm{FEV}}_{1}$ (${\%}_{\mathrm{pred}}$)	84 (26)	82 (26)	86 (27)	0.5
FVC (L)	3.6 (0.9)	3.4 (0.9)	3.9 (0.8)	**0.003**
FVC (${\%}_{\mathrm{pred}}$)	95 (17)	92 (16)	100 (17)	**0.03**
${\mathrm{FEV}}_{1}/\mathrm{FVC}$ (%)	65 (17)	66 (17)	63 (17)	0.5
TLC (L)	6.7 (1.3)	6.5 (1.3)	7.0 (1.2)	0.06
TLC (${\%}_{\mathrm{pred}}$)	109 (16)	108 (17)	111 (14)	0.4
RV/TLC (%)	43 (10)	45 (10)	42 (9)	0.2
${\mathrm{DL}}_{\mathrm{CO}}$ (${\%}_{\mathrm{pred}}$)	68 (21)	67 (21)	70 (23)	0.6
6MWD (m)	405 (81)	404 (84)	405 (75)	0.9
SGRQ	28 (21)	28 (20)	28 (23)	0.9

BMI, body mass index; ${\mathrm{SpO}}_{2}$, blood oxygen saturation; QoL, quality-of-life; ${\mathrm{FEV}}_{1}$, forced expiratory volume in 1 s; ${\%}_{\mathrm{pred}}$, percent of predicted value; FVC, forced vital capacity; RV, residual volume; TLC, total lung capacity; ${\mathrm{DL}}_{\mathrm{CO}}$, diffusing capacity of lung for carbon-monoxide; 6MWD, 6-min walk distance; and SGRQ, St. George’s respiratory questionnaire.

p = uncorrected values showing significant differences between $\Delta {\mathrm{FEV}}_{1}<60\;\mathrm{mL}/\mathrm{yr}$ and $\Delta {\mathrm{FEV}}_{1}\geq 60\;\mathrm{mL}/\mathrm{yr}$ groups. Significance level p<0.05.

**Table 2:** Participant demographics and pulmonary function measurements at follow-up visit.

Parameter mean (±SD)	All participants (n=88)	$\Delta {\mathrm{FEV}}_{1}<60\;\mathrm{mL}/\mathrm{yr}$ (n=57)	$\Delta {\mathrm{FEV}}_{1}\geq 60\;\mathrm{mL}/\mathrm{yr}$ (n=31)	p-value
Age	72 (9)	73 (9)	70 (9)	0.2
Female n (%)	29 (33)	22 (39)	7 (23)	0.1
Height (m)	169 (8)	168 (8)	170 (7)	0.2
BMI ($\mathrm{kg}/m^{2}$)	28 (4)	28 (4)	29 (5)	0.3
${\mathrm{SpO}}_{2}$ (%)	95 (3)	95 (4)	95 (2)	0.9
Pack years	36 (26)	37 (26)	35 (21)	0.6
Years since quit	17 (14)	17 (14)	17 (13)	0.9
Current smokers	3 (3)	2 (3)	1 (3)	0.9
Pulmonary function and QoL				
${\mathrm{FEV}}_{1}$ (L)	2.2 (0.8)	2.2 (0.8)	2.1 (0.7)	.4
${\mathrm{FEV}}_{1}$ (${\%}_{\mathrm{pred}}$)	84 (25)	87 (29)	77 (27)	.07
FVC (L)	3.3 (0.9)	3.3 (0.9)	3.4 (0.8)	.5
FVC (${\%}_{\mathrm{pred}}$)	94 (19)	95 (19)	92 (19)	.4
${\mathrm{FEV}}_{1}/\mathrm{FVC}$ (%)	65 (16)	66 (17)	62 (17)	.5
TLC (L)	6.4 (1.3)	6.3 (1.3)	6.6 (1.2)	.3
TLC (${\%}_{\mathrm{pred}}$)	105 (16)	105 (17)	106 (14)	.8
RV/TLC (%)	45 (10)	45 (10)	45 (9)	.7
${\mathrm{DL}}_{\mathrm{CO}}$ (${\%}_{\mathrm{pred}}$)	80 (21)	77 (25)	84 (28)	.6
6MWD (m)	398 (83)	396 (84)	400 (75)	.8
SGRQ	30 (21)	27 (20)	37 (23)	.08

BMI, body mass index; QoL, quality-of-life; ${\mathrm{SpO}}_{2}$, blood oxygen saturation; ${\mathrm{FEV}}_{1}$, forced expiratory volume in 1 s; ${\%}_{\mathrm{pred}}$, percent of predicted value; FVC, forced vital capacity; RV, residual volume; TLC, total lung capacity; ${\mathrm{DL}}_{\mathrm{CO}}$, diffusing capacity of lung for carbon-monoxide; 6MWD, 6-min walk distance; and SGRQ, St. George’s respiratory questionnaire.

p = uncorrected values showing significant differences between $\Delta {\mathrm{FEV}}_{1}<60\;\mathrm{mL}/\mathrm{yr}$ and $\Delta {\mathrm{FEV}}_{1}\geq 60\;\mathrm{mL}/\mathrm{yr}$ groups. Significance level p<0.05.

### 3.2 Imaging Measurements and Texture Features

Table 3 and Fig. S1 in the Supplementary Material summarize the texture feature definitions and descriptions used throughout the text. Table 4 summarizes quantitative MR imaging measurements and ranked texture features after feature selection step. Ex-smokers with accelerated lung function decline had significantly different local homogeneity normalized (Idmn, p=0.048) feature, large unventilated region emphasis (LGLZE, p=0.01) feature, small unventilated region emphasis (SRLGLE, p=0.007) feature, and local distribution of unventilated regions [small dependence low gray level emphasis (SDLGLE), p<0.001] feature. As summarized in Table 5, these subgroups did not have any statistically significant differences between imaging measurements at follow-up. Hyperpolarized gas MRI for representative participants in the stable and rapid decliner groups are shown in Fig. S1 in the Supplementary Material.

**Table 3:** MRI texture feature descriptors for machine-learning modeling.

Feature name	Short name	Description
Shape-major axis length	Longest distance across ventilated lung	Measures the largest axis length of the ellipsoid within the ROI, calculated using the voxel centers defining the ROI.
		It provides information about the elongation or stretching of shapes within the image, where a small value indicates that the shapes are less elongated or more compact along their major axis.
CDD1	No. of small ventilation defects	Measures the number of spheres with diameter of one voxel that can fit within the unventilated region of the lung, as visualized on hyperpolarized gas MR imaging.
		It provides information about the relative number of small ventilation defects and their clustering within the lung.
GLCM-Idn	Local homogeneity	Measures the local homogeneity of an image by normalizing the differences between the neighboring intensity values and dividing over the total number of discrete intensity values.
		It provides a measure of the overall smoothness or uniformity of texture patterns in an image, with higher values indicating a homogeneous texture.
GLCM-Idmn	Local homogeneity normalized	Measures the inverse difference moment of GLCM after it has been normalized by the sum of squared elements, which helps to mitigate the effects of image size and intensity variations.
		It provides information about the similarity of neighboring pixel values in the image. When the texture change in the image is not significant, the stronger the homogeneity of image information, the higher the value of Idmn.
LL-SZM-LGLZE	Large unventilated region emphasis	Measures how large areas of similar brightness or color are spread out across the image.
		It quantifies the distribution of large zones with low gray-level values within an image. A high value indicates that large zones with low gray-level values are prominent and spread out throughout the image.
LL-RLM-SRLGLE	Small unventilated region emphasis	Measures how short sequences of pixels with similar brightness or color and low gray-level values are distributed across the image.
		It quantifies the joint distribution of short runs with low gray-level values within an image. A high value indicates that short runs with low gray-level values are prominent in the image.
LL-GLDM-SDLGLE	Local distribution of unventilated regions	Measures the joint probability distribution of small dependences with low gray-level intensity values.
		It provides information about how frequently short runs with low gray-level values occur, as well as their spatial arrangement. A higher value indicates a greater emphasis on short runs with low gray-level values, suggesting the presence of localized dark regions or texture patterns in the image.

CDD1, cluster-defect diameter of one voxel; GLCM, gray level co-occurrence matrix; SZM, size zone matrix; RLM, run length matrix; GLDM, gray-level dependence matrix; Idn, inverse difference normalized; Idmn, inverse difference moment normalized; LL, low-low-pass filter; LGLZE, low gray level zone emphasis; SRLGLE, short run low gray level emphasis; and SDLGLE, short distance low gray level emphasis.

**Table 4:** Imaging measurements of participants with stable and accelerated lung function decline.

Parameter mean (±SD)	All Participants (n=88)	$\Delta {\mathrm{FEV}}_{1}<60\;\mathrm{mL}/\mathrm{yr}$ (n=57)	$\Delta {\mathrm{FEV}}_{1}\geq 60\;\mathrm{mL}/\mathrm{yr}$ (n=31)	p-value	p-value*
MRI VDP (%)	12 (9)	11 (8)	14 (11)	0.2	0.6
MRI ADC (${\mathrm{cm}}^{2}/s$)	0.34 (0.10)	0.33 (0.08)	0.36 (0.12)	0.1	0.4
ΔVDP (%)	4 (5)	3 (5)	4 (5)	0.9	0.9
ΔADC (${\mathrm{cm}}^{2}/s$)	0.02 (0.04)	0.02 (0.04)	0.02 (0.05)	0.3	0.6
**Selected texture features**					
Shape-major axis length	98.9 (7.5)	97.7 (7.7)	101.4 (7.1)	0.1	0.3
CDD1	4771 (3212)	4316 (2751)	5528 (3097)	0.1	0.3
GLCM-Idn	0.956 (0.008)	0.958 (0.008)	0.954 (.007)	0.095	0.3
GLCM-Idmn	0.995 (0.002)	0.995 (0.002)	0.997 (0.001)	**0.048**	0.2
Wavelet-filtered					
LL-SZM-LGLZE	0.00017 (0.00010)	0.00015 (0.00008)	0.00020 (0.00010)	**0.01**	**0.03**
LL-RLM-SRLGLE	0.00017 (0.00009)	0.00015 (0.00008)	0.00020 (0.00009)	**0.007**	**0.01**
LL-GLDM-SDLGLE	0.00016 (0.00007)	0.00014 (0.00006)	0.00019 (0.00007)	**<0.001**	**0.01**

MRI-VDP, ventilation defect percent; ADC, apparent diffusion coefficient; CDD1, cluster-defect diameter of one voxel; GLCM, gray level co-occurrence matrix; SZM, size zone matrix; RLM, run length matrix; GLDM, gray-level dependence matrix; LL, low-low-pass filter; Idn, inverse difference normalized; Idmn, inverse difference moment normalized; LGLZE, low gray level zone emphasis; SRLGLE, short run low gray level emphasis; and SDLGLE, small dependence low gray level emphasis.

p = uncorrected values showing significant differences between $\Delta {\mathrm{FEV}}_{1}<60\;\mathrm{mL}/\mathrm{yr}$ and $\Delta {\mathrm{FEV}}_{1}\geq 60\;\mathrm{mL}/\mathrm{yr}$ groups.

p* = Holm–Bonferroni corrected p-values. Significance level p<0.05.

Note: bold denotes statistically significant.

**Table 5:** Imaging measurements of participants with stable and accelerated lung function decline at follow-up visit.

Parameter mean (±SD)	All participants (n=88)	$\Delta {\mathrm{FEV}}_{1}<60\;\mathrm{mL}/\mathrm{yr}$ (n=57)	$\Delta {\mathrm{FEV}}_{1}\geq 60\;\mathrm{mL}/\mathrm{yr}$ (n=31)	p-value	p-value*
MRI-VDP (%)	16 (13)	15 (9)	18 (14)	0.1	0.4
MRI ADC (${\mathrm{cm}}^{2}/s$)	0.35 (0.11)	0.34 (0.07)	0.36 (0.10)	0.4	0.9
ΔVDP (%)	4 (5)	3 (5)	4 (5)	0.9	0.9
ΔADC (${\mathrm{cm}}^{2}/s$)	0.02 (0.04)	0.02 (0.04)	0.02 (0.05)	0.3	0.9
**Selected texture features**					
Shape-major axis length	97.9 (7.7)	97.5 (7.5)	99.8 (7.3)	0.6	0.6
CDD1	5037 (3866)	4616 (2951)	6128 (3298)	0.2	0.6
GLCM-Idn	0.956 (0.008)	0.954 (0.008)	0.957 (0.007)	0.3	0.6
GLCM-Idmn	0.995 (0.002)	0.995 (0.002)	0.996 (0.001)	0.1	0.4
Wavelet-filtered					
LL-SZM-LGLZE	0.00016 (0.00010)	0.00015 (.00008)	0.00019 (0.00010)	0.2	0.2
LL-RLM-SRLGLE	0.00016 (0.00010)	0.00015 (0.00008)	0.00019 (0.00010)	0.1	0.2
LL-GLDM-SDLGLE	0.00015 (0.00008)	0.00014 (0.00007)	0.00018 (0.00008)	0.07	0.2

MRI-VDP, ventilation defect percent; ADC, apparent diffusion coefficient; CDD1, cluster-defect diameter of one voxel, GLCM, gray level co-occurrence matrix; SZM, size zone matrix; RLM, run length matrix; GLDM, gray-level dependence matrix; LL, low-low-pass filter; Idn, inverse difference normalized; Idmn, inverse difference moment normalized; LGLZE, low gray level zone emphasis; SRLGLE, short run low gray level emphasis; and SDLGLE, short distance low gray level emphasis.

p = uncorrected values showing significant differences between $\Delta {\mathrm{FEV}}_{1}<60\;\mathrm{mL}/\mathrm{yr}$ and $\Delta {\mathrm{FEV}}_{1}\geq 60\;\mathrm{mL}/\mathrm{yr}$ groups.

p* = Holm–Bonferroni corrected p-values. Significance level p<0.05.

### 3.3 Machine learning Modeling

As summarized in Table 6, the best performing machine learning model trained on demographic measurements (age, sex, BMI, % ${\mathrm{SpO}}_{2}$, pack years, and years since quit) achieved 64% prediction accuracy. Best performing spirometry model (FVC, TLC, ${\mathrm{FEV}}_{1}$, ${\%}_{\mathrm{pred}}$ IC, SVC, and RV/TLC) was cosine KNN algorithm with 68% accuracy, which was not statistically different from the models based on demographics measurements. The texture-based model achieved the highest sensitivity (86%) and an 81% accuracy via the ensemble RUSBoosted trees algorithm, exclusively trained on selected MR image texture features. The combined model achieved the highest accuracy of 82% using the Medium-Gaussian SVM algorithm, trained on FVC, age, longest distance across ventilated lung, local homogeneity, large unventilated region emphasis, and local distribution of unventilated regions.

**Table 6:** Machine-learning performance at predicting accelerated lung function decline.

Best performing models	AUC	Sens. (%)	Spec. (%)	F1-score	Acc. (%)
**Demographics model** ^a^					
Logistic regression	0.64	66.7	46.2	75.7	63.6
**Spirometry model** ^b^					
Cosine KNN	0.68	68.9	63.6	79.1	68.2
**Texture-based model** ^c^					
RUSBoosted trees	0.80	**85.7**	71.9	85.5	80.7
**Combined model** ^d^					
Medium-Gaussian SVM	**0.81**	80.6	**85.7**	**87.1**	**81.8**

AUC, area under the receiver-operating curve; KNN, K-nearest neighbors; RUS, random under sampling; and SVM, support vector machine. *Bold values indicate highest performance in a specific metric.

a Variables used for training included: age, sex, BMI, % ${\mathrm{SpO}}_{2}$, pack years, and years since quit.

b Variables used for training included: FVC, TLC, ${\mathrm{FEV}}_{1}$, ${\%}_{\mathrm{pred}}$ IC, SVC, and RV/TLC.

c Features selected for training included: MRI cluster-defect diameter of one voxel (CDD1), shape-major axis length, gray level co-occurrence matrix-inverse difference normalized, gray level co-occurrence matrix-inverse difference moment normalized, wavelet-low-low-size zone matrix-low gray level zone emphasis, wavelet-low-low-run length matrix-short run low gray level emphasis, and wavelet-low-low-gray level dependence matrix-small dependence low gray level emphasis.

d Combined model included: FVC, sex, shape-major axis length, gray level co-occurrence matrix-inverse difference normalized, wavelet-low-low-size zone matrix-low gray level zone emphasis, wavelet-low-low- gray level dependence matrix-small dependence low gray level emphasis.

As summarized in Table 7, the machine learning models trained exclusively on MRI texture features outperformed the machine learning models trained using participant demographic and spirometry measurements (p<0.05). In addition, the combined model trained on all available measurements also outperformed the demographic and spirometry-based models (p<0.05); however, the performance of the combined model failed to show a significant difference (p=0.9) to the machine learning models trained exclusively on MRI texture features.

**Table 7:** DeLong’s test for comparing the models for predicting accelerated disease progression in ex-smokers.

Best model comparisons	p-value
Demographics model versus spirometry model	0.7
Demographics model versus texture-based model	**<0.001**
Demographics model versus combined model	**<0.001**
Spirometry model versus texture-based model	**0.04**
Spirometry model versus combined model	**0.03**
Texture-based model versus combined model	0.9

Significance level p<0.05.

Note: bold denotes statistically significant.

**Fig. 4:**
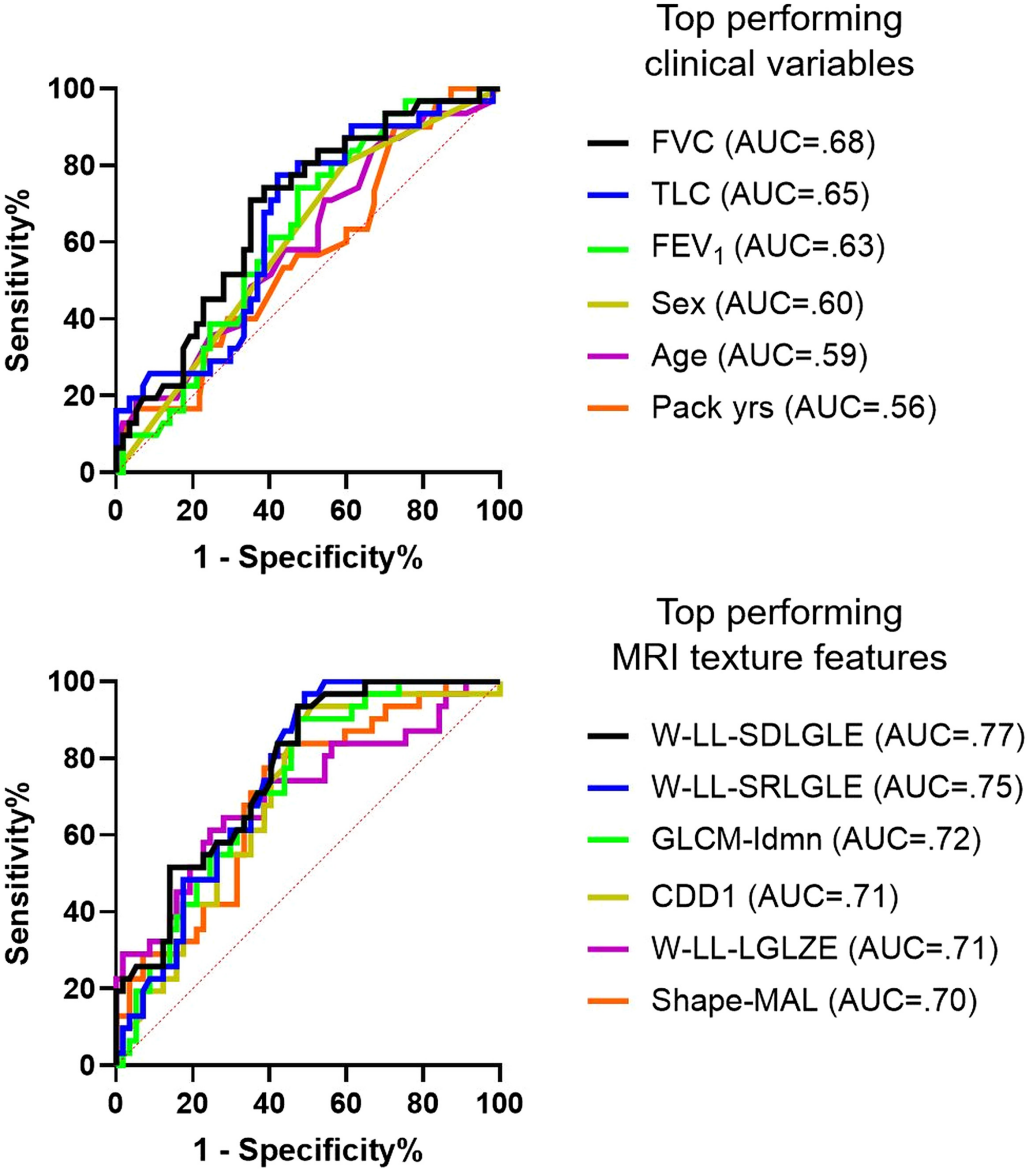
Receiver-operator characteristic curves of clinical and texture measurements. Top panel: logistic regression analysis of individual top-performing demographic and spirometry variables at predicting $\Delta {\mathrm{FEV}}_{1}\geq 60\;\mathrm{mL}/\text{year}$ in ex-smoker participants. Bottom panel: logistic regression of selected top-performing MR imaging and texture features at predicting $\Delta {\mathrm{FEV}}_{1}\geq 60\;\mathrm{mL}/\text{year}$. Individual texture features clearly outperformed established clinical variables available to physicians at predicting accelerated lung function decline. FVC, forced vital capacity; TLC, total lung capacity; ${\mathrm{FEV}}_{1}$, forced expiratory volume in 1 s; LL, low-low pass filter; SDLGLE, short distance low gray level emphasis; SRLGLE, short run low gray level emphasis; Idmn, inverse difference moment normalized; CDD1, cluster-defect diameter of one voxel; LGLZE, low gray level zone emphasis; and MAL, major axis length.

The ensemble models outperformed the single machine learning models, indicating the presence of more complex and non-linear relationships of texture features and accelerated lung function decline. Logistic regression models for predicting accelerated lung function decline were generated for individual clinical and imaging texture measurements with the receiver-operator characteristic curve AUC, which is summarized in Fig. 4. The best performing clinical measurements for predicting patients with accelerated ${\mathrm{FEV}}_{1}$ decline were FVC (AUC = 0.68) and TLC (AUC = 0.65). The overall best predictive measurement was wavelet-based local distribution of unventilated regions (SDLGLE, AUC = 0.77), which also outperformed standard imaging measurements, such as MRI VDP (AUC = 0.63).

### 3.4 Relationships with Clinical Measurements

**Fig. 5:**
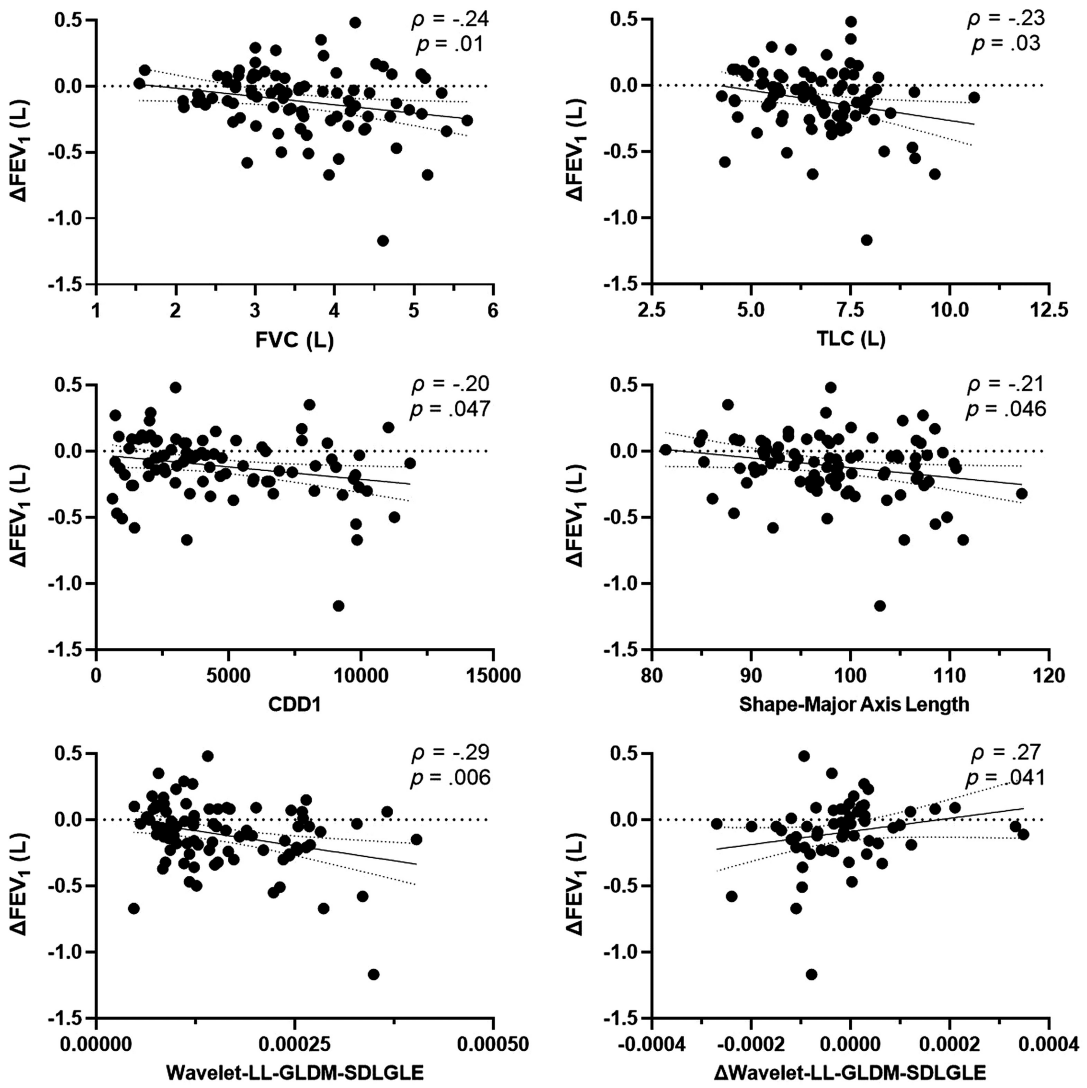
Relationships between selected texture features and change in FEV1.Top panel: Spearman correlation for FVC and TLC with $\Delta {\mathrm{FEV}}_{1}$ (ρ=−0.24, p=0.01; ρ=−0.23, and p=0.03, respectively) between baseline and follow-up visits. Middle panel: Spearman correlation for custom CDD1 feature and shape-major axis length with $\Delta {\mathrm{FEV}}_{1}$ (ρ=−0.20, p=0.047; ρ=−0.21, and p=0.046, respectively) between baseline and follow-up visits. Bottom panel: Spearman correlation for wavelet-LL-filtered-GLDM-SDLGLE and Δwavelet-LL-filtered-GLDM-SDLGLE with $\Delta {\mathrm{FEV}}_{1}$ (ρ=−0.29, p=0.006; ρ=0.27, and p=0.041, respectively) between baseline and follow-up visits. ${\mathrm{FEV}}_{1}$, forced expiratory volume in 1 s; FVC, forced vital capacity; TLC, total lung capacity; CDD1, cluster-defect diameter of one voxel; LL, low-low pass filter; GLDM, gray level dependence matrix; and SDLGLE, short distance low gray level emphasis.

Spearman correlations were used to evaluate the relationships between well-established clinical measurements and MRI texture features identified as significant predictors of clinically relevant ${\mathrm{FEV}}_{1}$ changes. As shown in Fig. 5, the best performing clinical measurements of FVC and TLC correlated with $\Delta {\mathrm{FEV}}_{1}$ between visits (ρ=−0.24, p=0.01; ρ=−0.23, and p=0.03, respectively). Similarly, texture features from the original unfiltered image representing the number of small ventilation defects and the longest distance across ventilated lung correlated with $\Delta {\mathrm{FEV}}_{1}$ (ρ=−0.20, p=0.047; ρ=−0.21, and p=0.046, respectively). The best performing wavelet-based local distribution of unventilated regions (SDLGLE) feature exhibited the strongest correlation with $\Delta {\mathrm{FEV}}_{1}$ (ρ=−0.29, p=0.006), and only the longitudinal change in this specific texture correlated with the clinically relevant changes in ${\mathrm{FEV}}_{1}$ (ρ=0.27, p=0.041).

## 4 Discussion

In this study, we developed an MRI texture analysis pipeline to reveal a subset of ventilation patterns that can help predict ex-smokers that will experience accelerated lung function decline. We observed that RUSBoosted trees algorithm trained solely on texture features outperformed all other existing models at predicting clinically relevant changes in ${\mathrm{FEV}}_{1}$ (81% accuracy). Overall, ensemble machine learning classifiers outperformed single classifiers, indicating the existence of complex non-linear relationships between ventilation patterns and lung function. Our findings suggest that texture-based features provide unique information about early functional changes occurring in the lungs, which may be used alongside established clinical measurements to identify ex-smokers at-risk of accelerated lung function decline.

We identified seven unique texture features residing within hyperpolarized He3 MR ventilation images in order to predict ex-smokers at risk of accelerated lung function decline. Standard MRI-derived measurements were outperformed by MRI texture features during the feature selection step. While model test accuracy was moderate, sensitivity remained high, which underscores the potential of this approach and hyperpolarized noble gas MRI. The values and equations^30^ of selected texture features represent large unventilated region emphasis, small unventilated region emphasis, and local distribution of unventilated regions within the lung. Collectively, these features can quantify the distribution of low intensity values or the clusters and sizes of poorly-ventilated lung regions. Local homogeneity (Idn) and local homogeneity normalized (Idmn) features quantify the inherent texture heterogeneity and can be thought of reflecting ventilation patchiness or non-uniformity within the lung. The novel extracted feature reflecting the number of small ventilation defects (CDD1) reflects the cumulative number of defect clusters of one voxel in size ($5\times 5\times 5\;{\mathrm{mm}}^{3}$) and describes defect clusters of low gray-level or signal void regions. Thus, MRI texture analysis provides quantitative information related to the patterns of gas distribution in the ventilated lung. In contrast, the proposed novel measurements analyze the unventilated regions of the lung, providing a holistic evaluation of the entire thoracic cavity volume on MRI.

Our results showed that MRI ventilation texture features were often selected as the most important features for predicting rapid lung function decline, even in the combined model. Previous studies have shown that CT radiomics features are associated with lung function in COPD,^7^ emphysema severity,^8^ and provide additional complementary information to established quantitative CT measurements.^9^ In more recent COPD studies, CT texture features were able to predict rapid lung function decline,^10^ whereas the combination of CT and MR imaging texture features were able to predict 10-year mortality risk.^39^ To our knowledge, this study is the first to show that MRI ventilation texture features predict accelerated lung function decline across a relatively short 3-year period. Compared to previous studies predicting a clinically relevant decline in ${\mathrm{FEV}}_{1}$ of ≥60  mL/year,^16^^,^^17^ our proposed model trained exclusively on MRI texture features exhibited a higher performance (AUC = 0.80) than existing clinical models by Lindberg et al. (AUC = 0.68)^40^ and CT radiomics-based models proposed by Makimoto et al. (AUC = 0.74).^10^ Furthermore, the best performing texture feature independently predicted and significantly correlated with longitudinal worsening in lung function. Interestingly, upon investigation, only the longitudinal changes in this specific MRI wavelet-based local distribution of unventilated regions feature coincided and correlated with longitudinal worsening in lung function. We showed that MRI texture features change along with changes in lung function and can differentiate rapid progressors, whereas previous work showed that MRI textures can also predict future mortality.^39^ Taken together, this suggests that MRI texture features offer unique information, not provided by established clinical measurements, and may serve as sensitive imaging biomarkers for early detection of patients at-risk of rapid worsening.

There were several study limitations. Our study included a relatively small sample size, and regardless of statistical techniques to prevent overfitting (univariate analysis, fivefold cross-validation, Boruta analysis, etc.), the machine learning classifiers could be optimized using larger datasets in the future. The generalizability could be further enhanced by incorporating an external dataset; thus, the generalizability of the machine learning models remains to be evaluated in future studies. Furthermore, three participants reported a change in smoking status from ex-smokers to current smokers at follow-up, with only one participant in the rapid decliner group and two participants in the stable decliner group. We acknowledge that smoking status is an important factor; however, in our study, it was not statistically different between groups and had minimal impact on results. Finally, the MR modality use in clinical settings is limited due to the availability and associated costs. Utility of hyperpolarized gas MRI is further limited due to additional personnel and equipment requirements. Therefore, although MRI-derived measurements provide unique prognostic value and are radiation-free, they are not nearly as readily available. However, with the recent FDA regulatory approval for the clinical use of Xe129 and associated equipment, we may see a shift in the near future in the utilization of MRI-derived measurements and biomarkers for evaluating lung diseases.

## 5 Conclusions

For the first time, machine learning and texture features from hyperpolarized He3 MRI ventilation images were used to predict ex-smokers who would experience accelerated ${\mathrm{FEV}}_{1}$ decline over a short 3-year period. Our work contributes to the growing body of evidence and is an important step for using imaging measurements to generate predictive models of lung function decline in ex-smokers with and without COPD.

## Supplementary Material



## Data Availability

Data generated or analyzed during the study are available from the corresponding author upon request.
