# Supplementary material for: Machine learning and magnetic resonance image texture analysis predicts accelerated lung function decline in ex-smokers with and without chronic obstructive pulmonary disease
Source: J Med Imaging (Bellingham). 2024 Jul 19;11(4):046001. doi: 10.1117/1.JMI.11.4.046001 (PMC11259551; doi:10.1117/1.JMI.11.4.046001)
Supplement: Supplementary file 1 [file JMI_011_046001_SD001.pdf]

Machine Learning and MR Image Texture Analysis Predicts Accelerated Lung Function Decline in Ex-smokers with and without COPD

Supplementary Material

Table S1. Texture analysis matrices and MRI features extracted from each ex-smoker participant

Category	Level/ Order	Description	Texture Features
Intensity histogram (n=19)	First-order	Distribution of pixel or voxel intensities within the image ROI defined by the mask	Energy, Total energy, Entropy, Minimum, Maximum, 10th, 90th percentile, Mean, Median, Interquartile range, range, MAD, rMAD, RMS, SD, Skewness, Kurtosis, Variance, Uniformity
Shape-based (n=26)	First-order	2D and 3D size and shape features of the ROI; independent of the pixel or voxel gray level intensity distribution	Mesh volume, Mesh surface, Pixel surface, Perimeter, Perimeter to surface, Surface to volume, Voxel volume, Surface area, SAV, sphericity, Compactness, Spherical disproportion, M2DD, M3DD, M2DDS, M2DDC, M2DDR, Elongation, Flatness, Minor, Major, and Least axis length
GLRLM (n=16)	Second-order	Quantifies consecutive pixels or voxels of the same gray level in a given direction	SRE, LRE, GLN, GLNN, RLN, RLNN, RP, GLV, RV, RE, LGLRE, HGLRE, SRLGLE, SRHGLE, LRLGLE, LRHGLE
GLCM (n=24)	Second-order	Examines the spatial relationship among pixels and defines how frequently pairs of pixels are present in an image in a given direction	Autocorrelation, Joint average, Cluster prominence, Cluster shade, Cluster tendency, Contrast, Correlation, Difference average, Difference entropy, Difference variance, Joint energy, Joint entropy, IMC1, IMC2, IDM, MCC, IDMN, ID, IDN, Inverse variance, Maximum probability, SA, SE, SS
GLSZM (n=16)	Second-order	Quantifies the number of homogeneous connected voxels that share the same gray level intensity in an image	SAE, LAE, GLN, GLNN, SZN, SZNN, ZP, GLV, ZV, ZE, LGLZE, HGLZE, SALGLE, SAHGLE, LALGLE, LAHGLE
NGTDM (n=5)	Second-order	Quantifies the difference between a gray value and the average gray value of its neighbours within a specific distance and direction	Coarseness, Contrast, Busyness, Complexity, Strength
GLDM (n=14)	Second-order	Quantifies the number of connected voxels within a specific distance that are dependent on the center voxel in the ROI of an image	SDE, LDE, GLN, GLNN, DN, DNN, GLV, DV, DE, LGLE, HGLE, SDLGLE, SDHGLE, LDLGLE, LDHGLE
Wavelet-based (n=376)	Higher-order	Applies combinations of high-pass and low-pass wavelet filters to the input image axes to yield the space-frequency decompositions	Four high/low-pass filter combinations applied across the image; Excluding the 16 Shape-based features: $4\times(19+16+24+16+5+14) = 376$

n=number of unique extracted texture features; GLRLM=Gray-level Run Length Matrix; GLCM=Gray-level Co-occurrence Matrix; GLSZM= Gray-level Size-zone Matrix; NGTDM= Neighbourhood Gray Tone Difference Matrix; GLDM=Gray-level Dependence Matrix; MAD=Mean Absolute Deviation; rMAD=Robust MAD; RMS=Root Mean Squared; SD=Standard deviation; SAV=Surface area to volume ratio; M3DD=Maximum 3D diameter; M2DDS=Maximum 2D diameter Slice; M2DDC=M2DD Column; M2DDR=M2DD Row; SRE=Short Run Emphasis; LRE=Long Run Emphasis; GLN=Gray Level Non-Uniformity;

GLNN=GLN Normalized; RLN=Run Length Non-Uniformity; RLNN=RLN Normalized; RP=Run Percentage; GLV=Gray Level Variance; RV=Run Variance; RE=Run Entropy; LGLRE=Low Gray Level Run Emphasis; HGLRE=High Gray Level Run Emphasis; SRLGLE=Short Run Low Gray Level Emphasis; SRHGLE=Short Run High Gray Level Emphasis; LRLGLE=Long Run Low Gray Level Emphasis; LRHGLE=Long Run High Gray Level Emphasis; IMC=Informational Measure of Correlation; IDM=Inverse Difference Moment; MCC=Maximal Correlation Coefficient; IDMN=IDM Normalized; ID=Inverse Difference; IDN=ID Normalized; SA=Sum Average; SE=Sum Entropy; SS=Sum of Squares; SAE=Small Area Emphasis; LAE=Large Area Emphasis; SZN=Size-Zone Non-Uniformity; SZNN=SZN Normalized; ZP=Zone Percentage; ZV=Zone Variance; ZE=Zone Entropy; LGLZE=Low Gray Level Zone Emphasis; HGLZE=High Gray Level Zone Emphasis; SALGLE=Small Area Low Gray Level Emphasis; SAHGLE=Small Area High Gray Level Emphasis; LALGLE=Large Area Low Gray Level Emphasis; LAHGLE=Large Area High Gray Level Emphasis; SDE=Small Dependence Emphasis; LDE=Large Dependence Emphasis; DN=Dependence Non-Uniformity; DNN=DN Normalized; DV=Dependence Variance; DE=Dependence Entropy; LGLE=Low Gray Level Emphasis; HGLE=High Gray Level Emphasis; SDLGLE=Small Dependence Low Gray Level Emphasis; SDHGLE=Small Dependence High Gray Level Emphasis; LGLGLE=Large Dependence Low Gray Level Emphasis; LDHGLE=Large Dependence High Gray Level Emphasis.

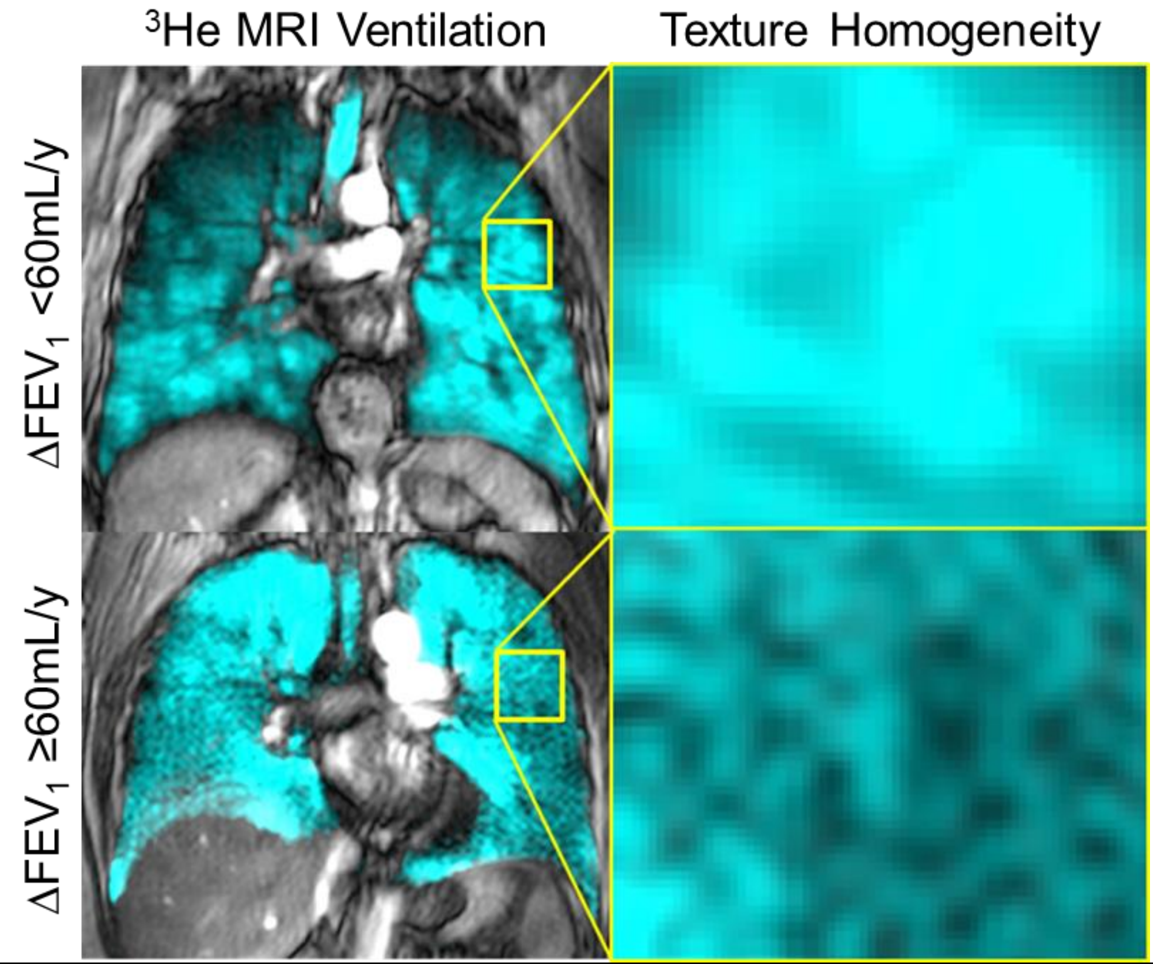

Parameter mean(±SD)	ΔFEV$_1$ <60mL/y	ΔFEV$_1$ ≥60mL/y
VDP (%)	5 %	5 %
ADC cm^2/s	0.24	0.26
Shape-Major Axis Length	96.6	101.7
CDD1	2287	5225
GLCM-Idn	0.959	0.955
GLCM-Idmn	0.994	0.997
LL-SZM-LGLZE	0.00014	0.00020
LL-RLM-SRLGLE	0.00015	0.00019
LL-GLDM-SDLGLE	0.00014	0.00019

Figure S1. Hyperpolarized gas MRI for representative participants from stable and accelerated FEV$_1$ decline subgroups.

Top panel: A 66 year-old female ex-smoker with stable FEV$_1$ decline: FEV$_1$=80%$_{pred}$, FEV$_1$/FVC=76, BMI=36 kg/m^2, VDP=5.4%, and visually homogeneous ventilation textures.

Bottom panel: A 64 year-old female ex-smoker with accelerated FEV$_1$ decline: FEV$_1$=111%$_{pred}$, FEV$_1$/FVC=82, BMI=36 kg/m^2, VDP=4.6%, and visually heterogeneous ventilation textures.
